# Fibroblasts profiling in scarring trachoma identifies IL-6 as a functional component of a fibroblast-macrophage pro-fibrotic and pro-inflammatory feedback loop

**DOI:** 10.1038/srep28261

**Published:** 2016-06-20

**Authors:** Jenny Z. Kechagia, Daniel G. Ezra, Matthew J. Burton, Maryse Bailly

**Affiliations:** 1 Department of Cell Biology, UCL Institute of Ophthalmology, 11-43 Bath Street, London, EC1V 9EL, UK; 2 Moorfields Eye Hospital, and UCL Partners AHSC, 11-43 Bath Street, London, EC1V 9EL, UK; 3 International Centre for Eye Health, London School of Hygiene & Tropical Medicine, Keppel Street, London, WC1E 7HT, UK

## Abstract

Trachoma is a conjunctiva scarring disease, which is the leading infectious cause of blindness worldwide. Yet, the molecular mechanisms underlying progressive fibrosis in trachoma are unknown. To investigate the contribution of local resident fibroblasts to disease progression, we isolated conjunctival fibroblasts from patients with scarring trachoma and matching control individuals, and compared their gene expression profiles and functional properties *in vitro*. We show that scarring trachoma fibroblasts substantially differ from control counterparts, displaying pro-fibrotic and pro-inflammatory features matched by an altered gene expression profile. This pro-inflammatory signature was exemplified by increased IL-6 expression and secretion, and a stronger response to macrophage-mediated stimulation of contraction. We further demonstrate that scarring trachoma fibroblasts can promote Akt phosphorylation in macrophages in an IL-6 –dependent manner. Overall this work has uncovered a distinctive molecular fingerprint for scarring trachoma fibroblasts, and identified IL-6- as a potential contributor to the chronic conjunctival fibrosis, mediating reciprocal pro-fibrotic/pro-inflammatory interactions between macrophages and fibroblasts.

Despite being one of the earliest diseases ever described, trachoma remains the leading infectious cause of blindness worldwide and the pathophysiological basis for this progressive fibrotic disease remains an unresolved enigma. The World Health Organization currently estimates 231 million people live in trachoma endemic areas, with 2.2 million being visually impaired^1^. Trachoma is caused by infection with *Chlamydia trachomatis,* which initially leads to conjunctival inflammation (active disease). Repeated re-infections can cause chronic inflammation, which can lead to scarring, whereby the eyelid turns inwards (entropion) and the eyelashes rub on the cornea (trichiasis). This leads to corneal opacification and eventually blindness^2^. While the active disease appears to affect mainly children, scarring trachoma is more common in adulthood, often developing decades after childhood disease and in the absence of detectable *C. trachomatis*^3^. Although the early infection stages are fairly well characterised^4^,^5^, little is known about why and how scarring develops, and no suitable animal model is available^6^.

Scarring consequences in trachoma are characterized by the presence of a highly fibrotic conjunctiva/tarsal plate with increased matrix deposition and a compact a-vascular stroma, mainly composed of fibroblasts and inflammatory cells^7^. Despite the well-characterized role of fibroblasts in scarring and fibrosis, little is known about their contribution to progressive scarring in trachoma. A recent study comparing gene expression profiles in conjunctival swabs from control individuals and patients with trachomatous trichiasis revealed an enrichment in immune response mediators and keratinization factors^8^. However, such samples are confined to the conjunctiva outer layers and contain mixed cell populations, so may not accurately reflect changes occurring in the substantia propria –stroma– where the fibroblasts lie.

Here we describe the phenotypic and genomic profile of fibroblasts isolated from conjunctival biopsies collected from Tanzanian individuals with scarring trachoma and from control subjects from the same area. We show that trachoma-derived fibroblasts display a classical pro-fibrotic phenotype, which is underlined by a specific gene expression profile. In addition, we identify IL-6 as a signature gene for scarring trachoma, and demonstrate that its main role is in modulating the local immune response, potentially contributing to the chronic activation of the fibroblasts.

## Results

### Trachoma-derived fibroblasts exhibit a higher ability to contract 3D collagen matrices

**Figure 1:**
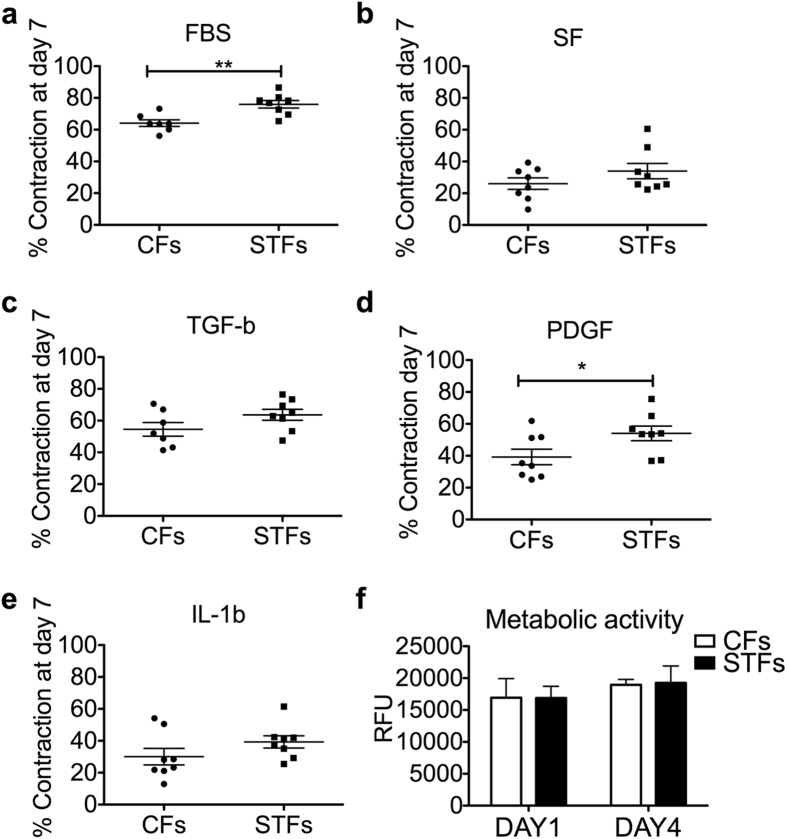
Scarring trachoma fibroblasts display increased matrix contraction abilities. Control (CFs) and trachoma-derived (STFs) fibroblasts were embedded in collagen matrix and contraction was measured over a 7-day period, in medium with 10% serum (FBS), serum-free medium (SF), or SF with cytokines (PDGF-BB 10 μg/mL, TGF-b1 5 μg/mL and IL-1b 10 ng/mL). **(a–e)** STFs exhibited higher contraction compared to CFs in the presence of serum (**p = 0.0025, two-tailed t-test), or PDGF-BB (*p = 0.0438). Shown is mean +/− SEM for gel contraction at day 7 (each data point as the average of 3–4 experiments with triplicate gels for 8 STFs and 8 CFs). **(f)** CFs and STFs have similar metabolic activity at days 1 and 4 of contraction, as measured with an Alamar blue fluorescence assay (mean +/− SEM for n = 3 for 4 CFs and 4 STFs).

Primary fibroblast cell lines were established from trachomatous trichiasis (Scarring Trachoma Fibroblasts, STFs) and matching control conjunctival biopsies (Control Fibroblasts, CFs) with a similar success rate (12/26 and 10/20 for trachomatous trichiasis and control biopsies respectively, with similar age range and gender balance; SI Appendix, Table S1). There were no obvious morphological differences between the two sets (Fig. S1a) and they displayed comparable integrin expression profiles, suggesting a similar tissue origin (Fig. S1b). The primary fibroblasts cultures were expanded and frozen in aliquots to generate substantial stocks of cells at low passage. A subset of 8 control (C24, C26, C29, C30, C31, C103, C110, C111) and 8 trachoma (F07, F09, F10, F11, F12, F99, F106, F109) fibroblast cultures, for which we had the largest stocks, were chosen for further analysis. We used our standard *in vitro* collagen gel contraction assay^9^,^10^ to determine the contractile profile of STFs and CFs, in the presence of serum (10%) or cytokines and growth factors previously linked to active and scarring trachoma (PDGF-BB, TGF-b1, IL-1b, IL-17A, TNFα, CTGF, CXCL5)^2^,^8^. STFs were on average more contractile than CFs in all conditions, although this trend was only significant for serum and PDGF stimulation (Fig. 1a–e). Both STFs and CFs responded well to TGFb1 and PDGF, but barely to IL-1b (Fig. 1c–e), and none of the other cytokines tested had any effect on contraction (not shown). There was no difference in cell viability/proliferation between CFs and STFs during contraction in the presence of serum (Fig. 1f), suggesting that the increased contraction ability displayed by STFs is an intrinsic cell feature and not a consequence of altered proliferation and/or survival.

### Trachoma-derived fibroblasts display a mildly altered matrix remodelling phenotype

**Figure 2:**
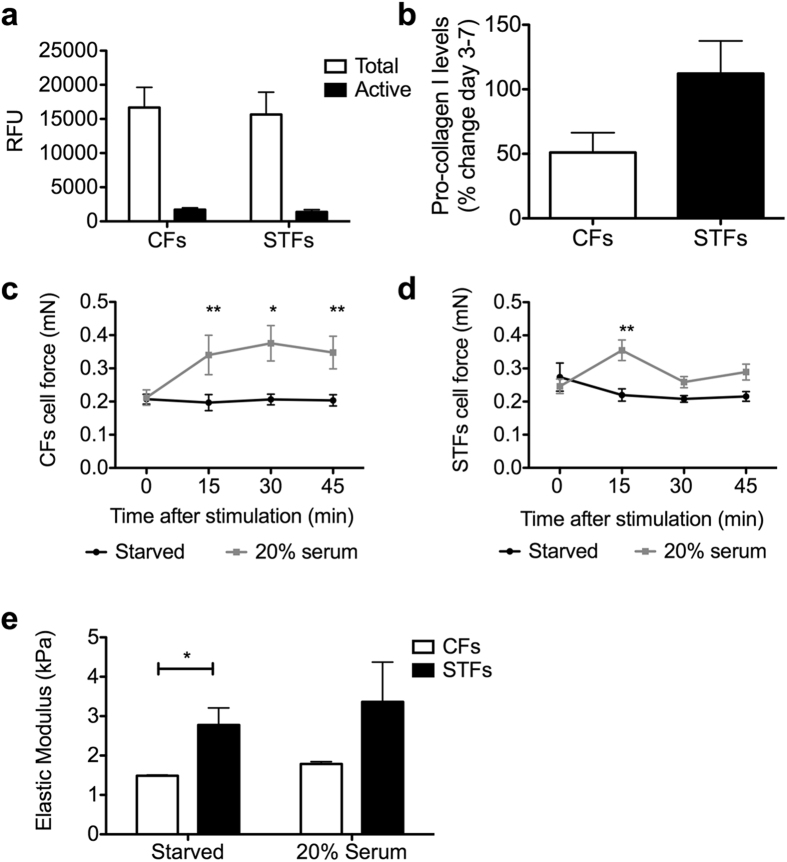
Scarring trachoma fibroblasts display altered force responses and matrix remodelling properties. **(a,b)** CFs and STFs (8 different cell lines each) were embedded in collagen gels for contraction in the presence of 10% serum and the culture medium was collected on days 3 and 5 for measurement of **(a)** MMP release (Active fraction and total amount following APMA activation), and **(b)** and day 3 to day 7 for neo-collagen synthesis. Shown are the means +/− SEM of 8 CFs and 8 STFs (n = 2–3 for each cell line). **(c–e)** CFs **(c)** and STFs **(d)** were embedded in collagen gels in MC-8™ micro-chambers and cultured for 2 days to generate tissues. The tissues were starved for 48 hours and cellular force was measured every 15 min after the addition of 20% serum. A mock stimulated control set (Starved) was done in parallel for each experiment. Shown is cell force, with mean +/− SEM for n = 1–3 for 8 CFs and 8 STFs (**<0.01, *<0.05, significantly different from time 0, 2-way ANOVA, Benferroni post-test). **(e)** Force measurements were acquired for CFs (C24, C30, C31) and STFs (F10, F11, F12) as in **(c,d)** above and the Elastic modulus (*E*) of the tissues was extracted from the matrix force formula *F*_*m*_ = *E x A*, where *A* is the tissue cross-section area. Shown is the mean+/− SEM (n = 4 for each cell line with 3 time points upon stimulation; *p = 0.0152, two-tailed t-test).

Fibrotic tissue is characterized by increased matrix remodelling including both neo-matrix synthesis and matrix degradation, and we have shown that matrix remodelling is an essential component of fibroblast-mediated gel contraction^11^,^12^. We thus sought to determine whether exaggerated matrix remodelling properties could underlie STF contractile phenotype. Control and trachoma fibroblasts displayed similar morphological features in collagen gels during contraction (Fig. S2). Confocal reflection microscopy revealed a small, but consistent, increase in matrix degradation in gels populated with STFs, with a more hazy appearance of the collagen fibres, reflecting some level of alteration (Fig. S2)^12^. However, there was no difference in the total amount or active proportion of soluble matrix metalloproteinases (MMPs) released by the cells during contraction (Fig. 2a). Similarly, although collagen type I neo-synthesis was higher on average for STFs, the difference was not statistically significant (p-value = 0.0585; Fig. 2b).

### Trachoma-derived fibroblasts display altered contraction force and tissue mechanics

Alpha smooth-muscle actin (a-SMA) expression is one of the most well characterized markers of fibroblast activation towards the contractile myofibroblast phenotype commonly encountered in fibrosis. Using immunofluorescence to analyse a-SMA expression during contraction, we could not detect any a-SMA expression at day 1 of culture. The proportion of a-SMA- positive fibroblasts increased thereafter to about 40–50% a-SMA positive cells at day 7. Again, although STFs had on average slightly more a-SMA positive cells, the difference was not significant (Fig. S3). We have shown previously a link between fibroblasts’ contraction potential and intrinsic cellular force levels^9^,^13^. To determine whether STFs displayed altered cellular force, we used the Palpator^TM^ (InvivoSciences, USA), an automated force measurement platform, to measure cellular contractile force and matrix stiffness in pre-stressed 3D collagen hydrogels populated with STFs and CFs. The cells were embedded in collagen gels in customized supports allowing gel contraction under tensioned conditions, leading to the formation of a tissue-like structure where the cells establish tensional homeostasis^14^. There was no difference in the ability of STFs and CFs to generate tissue constructs, and they displayed similar levels of cell force at resting state (after 48 hours starvation in serum-free medium, Fig.2c,d). Likewise, both cell types displayed a significant and comparable increase in cell force (by about 40%, p < 0.01) upon stimulation with 20% serum. However, while the force remained stable for CFs for up to 45 min (Fig. 2C), it was only transient in STFs, suggesting altered cellular force responses (Fig. 2d).

To further explore the mechanical properties of the tissue constructs, we used the matrix force properties acquired with the Palpator^TM^ to extract the elastic modulus (E), after calculating the cross sectional area of the collagen constructs^14^. We found that tissue constructs generated by CFs and STFs had levels of stiffness between 1 and 4 kPa, within the range of that of intact connective tissue^15^. However, tissues generated by STFs were significantly stiffer, with an Elastic Modulus value of about twice that of the tissues generated by CFs (Fig. 2e).

### Trachoma-derived fibroblasts display a specific pro-inflammatory and pro-fibrotic gene expression profile

**Figure 3:**
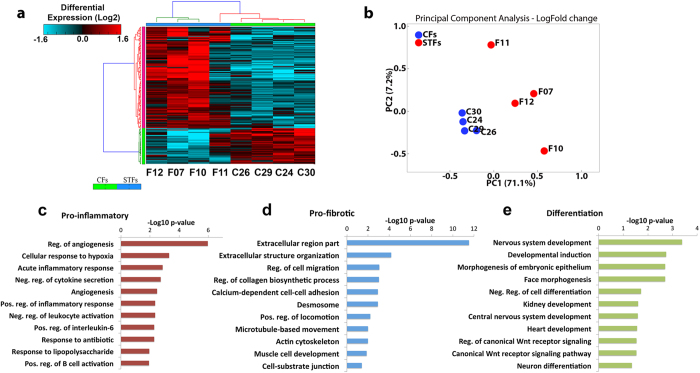
Scarring trachoma fibroblasts display a distinct gene expression profile. **(a)** Heat map of transcriptional changes in 187 protein-coding genes in 4 CFs (C26, C29, C24, C30) and 4 STFs (F12, F07, F10, F11). Genes were clustered using hierarchical average linkage clustering and euclidean distances. The vertical and horizontal bars adjacent to the heatmaps are coloured based on a flat cluster threshold of 0.7 (distance criterion). **(b)** Principal component analysis shows close clustering of CFs whilst STF samples are more variable. **(c–e)** Gene ontology analysis of differentially expressed genes, as predicted by GO-Elite (z-score >2, Fisher’s exact p-value <0.05).

To identify molecular markers associated with the pro-fibrotic profile of STFs, we performed a comparative gene expression analysis of STFs and CFs using a microarray platform (Affymetrix human gene 1.0 ST). Data analysis was done using AltAnalyze (http://www.altanalyze.org/), and the raw Affymetrix CEL files were processed using the Robust Multi-array Average (RMA) normalization methodology^16^. Due to the small sample size, we conducted a moderate t-test, as calculated by limma’s R package. We found that 128 genes were upregulated and 46 were downregulated in STFs compared to CFs, exhibiting more than 2 fold change (FC) and P values <0.05 (Table S3). Figure 3A illustrates the hierarchical clustering of gene expression patterns shared by the samples. The principal component analysis confirmed tight clustering of the control samples (Fig. 3b). There was more variability within the trachoma samples, with F11 in particular clustering closer to controls.

**Figure 4:**
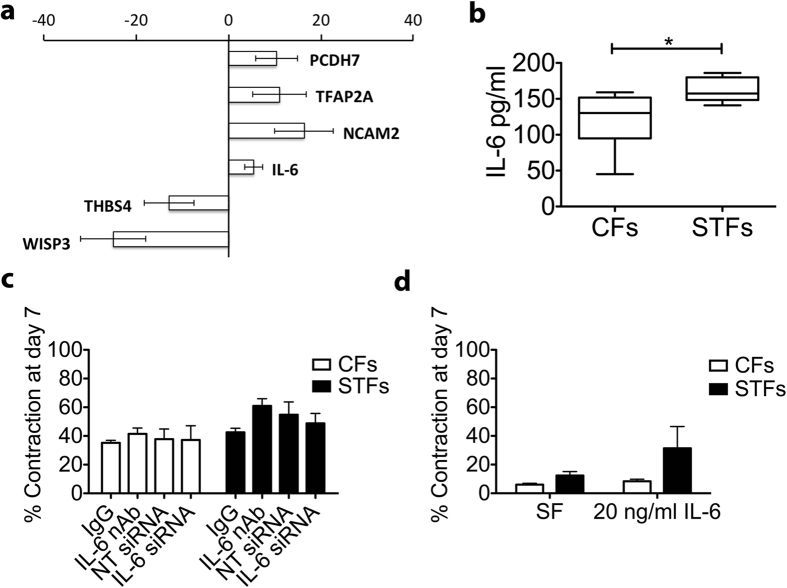
IL-6 is overexpressed in scarring trachoma fibroblasts but does not directly affect matrix contraction. **(a)** Sample genes from the microarray were validated using quantitative real-time PCR using RNA extracted from 6 CFs (C03, C24, C29, C30, C103, C111) and 7 STFs (F07, F09, F109, F99, F11, F106, F109) (*n* = 2–3 repeats each). Results are expressed as fold change over CFs samples, following normalization to GAPDH expression (mean +/− SEM). **(b)** CFs and STFs were embedded in collagen gels and allowed to contract in the presence of 10% serum. IL-6 levels were measured at day 7 using ELISA. Graph shows the mean +/− SEM for 7 CFs and 8 STFs cell lines (n = 2 for each; *p = 0.0249; two-tailed t-test). **(c)** Inhibition of IL-6 using neutralizing antibody (IL-6 nAb; 300 ng/ml) or siRNA (IL-6 siRNA) did not alter CFs or STFs contraction compared to control immunoglobulin (IgG) or Non-target control siRNA (NT-siRNA) respectively. Graph shows the mean+/− SEM for contraction at day 7 in presence of 10% serum (n = 2–3 for 4 CFs and 5 STFs). **(d)** IL-6 (20 ng/ml) did not significantly stimulate contraction. Graph shows mean+/− SEM for gel contraction at day 7, in serum free (SF) medium (n = 2 for 4 CFs and 4 STFs).

GO-Elite software was used to identify a non-redundant set of ontology terms and pathways enriched in STFs^17^. Gene ontology revealed genes associated with extracellular matrix organization, positive regulation of inflammatory response, developmental process, and Wnt signalling. Overall, the molecular processes enriched in our dataset can be divided into three different categories: pro-inflammatory, pro-fibrotic and genes associated with cell differentiation and development (Fig. 3c,d, Table S4). In addition, pathway analysis highlighted the involvement of cell differentiation processes and enrichment of genes related with TGF-b pathway, drawing a pro-fibrotic gene expression profile (Table S6). Using RT-qPCR analysis, we validated the differential expression of 6 of the most affected genes (NCAM2, TFAP2A, IL-6, PCDH7, THBS4 and WISP3), confirming a significantly altered genomic expression profile in STFs (Fig. 4a).

### IL-6 production by trachoma-derived fibroblasts contributes to macrophage activation

Amongst the genes identified as differentially expressed in STFs and implicated in most pathways, particularly noticeable was the prominent place of *IL6* (Fig. 3, Tables S3 and S4). IL-6 is an important mediator of inflammation and a previously suggested risk factor for scarring trachoma^18^. *IL6* expression was found to be increased 6 fold in STFs compared to CFs in the microarray study, and this increase was validated using real time qPCR (Fig. 4a). An ELISA analysis of the medium during contraction in the presence of 10% serum confirmed significantly higher IL-6 levels for STFs (Fig. 4b). To investigate whether this increased IL-6 expression could be linked to STFs’ contractile potential, we used blocking antibodies and siRNA to neutralise or prevent IL-6 release during contraction. Despite significantly blocking IL-6 function and expression respectively (Fig. S4a), neither antibody nor siRNA treatment affected CFs or STFs contraction (Fig. 4c). Likewise, IL-6 did not substantially stimulate contraction (Fig. 4d).

**Figure 5:**
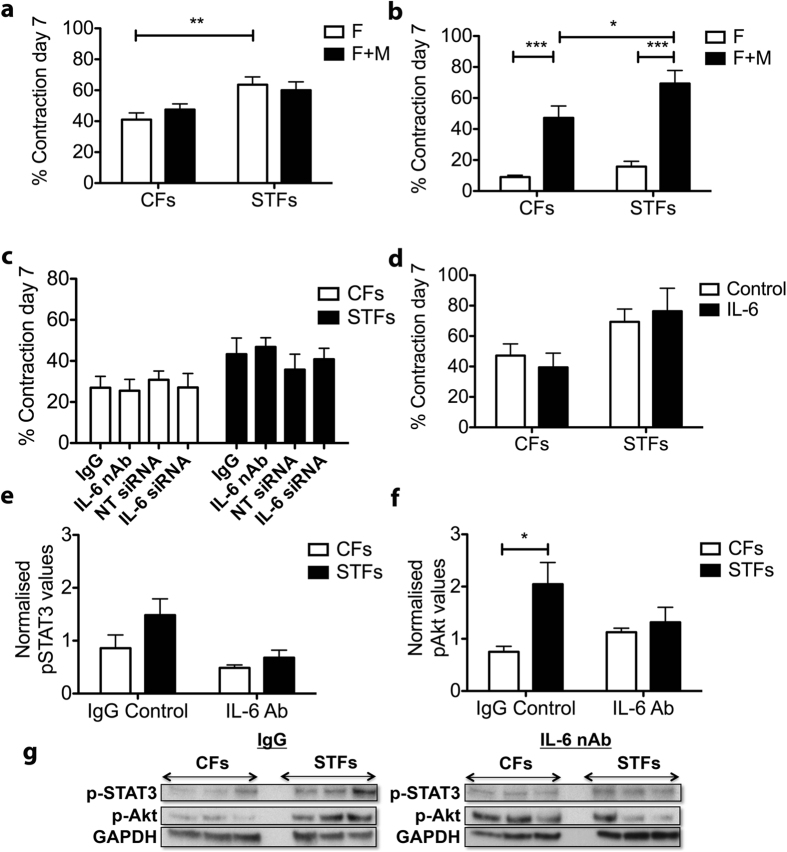
IL-6 production by scarring trachoma fibroblasts contributes to macrophage activation. Fibroblasts alone (F) or fibroblasts with macrophages (F+M) at a 1:4 ratio were embedded in collagen gels and contraction was allowed to proceed for 7 days in the presence of 10% serum (**a**) or in serum free medium **(b–d)** with/without IL-6 inhibition or soluble recombinant IL-6. (**a**) Macrophages do not affect fibroblast-mediated contraction in the presence of serum. Shown is the mean+/− SEM for contraction at day 7 (n = 2–3 for 4 CFs and 5 STFs; **p = 0.0002, t-test). (**b**) Fibroblast and macrophage co-culture in the absence of serum led to a significant increase in contraction for both CFs and STFs (p = 0.0013 and p = 0.0002, 2-tailed t-test), and higher for STFs (*p = 0.030, non-parametric t-test; n = 2–3 for 5 CFs and 5 STFs). (**c**) IL-6 inhibition in co-culture, either with neutralizing antibody (nAb, 300 ng/ml) or siRNA treatment against IL-6, did not alter contraction (mean+/− SEM for 1–3 experiments for 4 CFs and 5 STFs). (**d**) Addition of IL-6 (20 ng/ml) did not alter contraction in co-cultures (mean+/− SEM for n = 1-3 for 4 CFs and 6 STFs). (**e–g**) Condition medium (CM) was generated from CFs and STFs cultures and IL-6 nAb or control IgG (200 ng/ml) was added to it prior to adding it to macrophages. After 24 h incubation the cells were lysed and pathway activation was tested by western blot. Macrophages treated with STF-derived CM exhibited an increased activation of STAT3 (**e**) and Akt pathways (**f**), which was significantly reduced upon IL-6 inhibition. Graphs show protein levels relative to GAPDH (**p = 0.0129, two-tailed t-test; n = 4 for 4 CFs and 5 STFs). (**g**) Representative corresponding western blots.

Scarring in trachoma is often characterised by an increased inflammatory infiltrate, including macrophages and lymphocytes^19^. Owing to the strong involvement of IL-6 in the pro-inflammatory expression profile of STFs, we hypothesized that the increase IL-6 expression in STFs might be linked to inflammation. We produced differentiated macrophages from the U937 monocyte human cell line using PMA stimulation, and co-cultured them with CFs or STFs within collagen gels. Macrophages alone did not induce any contraction (Fig. S4b), and in the presence of 10% serum did not affect fibroblast-mediated gel contraction, even at a fibroblast:macrophage ratio of 1:4 (Fig. 5a). However, in the absence of serum, macrophages strongly stimulated fibroblast-mediated collagen contraction (Fig. 5b). As for serum conditions, macrophages alone did not cause any contraction (Fig. S4b). Importantly, the increase in contraction upon co-culture with macrophages was higher for STFs compared to CFs (p < 0.05). To test whether this difference could be linked to the increased IL-6 production in STFs, we examined the contraction potential of fibroblast:macrophage co-cultures in the presence of IL-6 neutralizing antibody, or using fibroblasts treated with IL-6 siRNA. Again, neither blocking/downregulating IL-6 (Fig. 5c), nor stimulating with IL-6 (Fig. 5d), affected fibroblast:macrophage co-cultures, suggesting that IL-6 does not directly mediate macrophages’ stimulation of the contraction. We then treated macrophages with condition medium from CFs and STFs, with or without the addition of IL-6 neutralising antibodies or corresponding control IgG, to determine whether IL-6 could modulate macrophage activation. The main signal transduction pathways triggered upon IL-6 stimulation involves the phosphorylation of signal transducer and activator of transcription 3 (STAT3) and Akt (or Protein kinase B [PKB]), responsible for the pro-survival signalling of IL-6^20^. Accordingly, recombinant IL-6 triggered Akt and STAT3 phosphorylation in U937-derived macrophages (Fig. S4c). Similarly, STF conditioned medium increased both STAT3 (Fig. 5e,g) and Akt (Fig. 5f,g) phosphorylation in macrophages compared to CF-conditioned medium, although the difference was only significant for Akt phosphorylation because of greater intercellular variability in STAT3 phosphorylation levels. This increase in phosphorylation was prevented when IL-6 was neutralized in the conditioned medium prior to addition to the macrophages, leading to phosphorylation levels comparable to those obtained following treatment with CF-conditioned medium. Similar results were obtained when using conditioned medium from fibroblasts treated with IL-6 siRNA (Fig. S4d,e), indicating that IL-6 derived from STFs can trigger signalling activation in macrophages.

## Discussion

Trachoma is a major cause of blindness worldwide^1^. Despite being one of the oldest diseases ever recorded, our knowledge of the mechanism of scar tissue formation, responsible for the devastating blinding consequences, is still limited. While this may be partly attributed the lack of a suitable animal model, the research so far has focused on the complex immune responses, with little attention being paid to the local tissue stroma. Previous studies, using conjunctival swabs from patients with trichiasis, have identified a number of genes potentially underlying the fibrotic phenotype in trachoma^8^. These included keratinization markers and classical inflammation components, such as *IL1B*, *CXCL5*, *INDO* and *S100A7*, as well as MMPs. However, such samples are often inflamed and contain a large fraction of the epithelium and immune cells, making it difficult to identify the contribution of the underlying fibroblasts. Previous work from our group^9^ and others^21^ have shown that fibroblasts isolated from ocular fibrotic tissue retain pro-fibrotic features and distinct genomic profiles *in vitro*, allowing the identification of putative novel disease pathways. Here we used a similar *in vitro* system to characterise some of the key features of scarring trachoma fibroblasts and investigate their contribution to disease progression. We found that trachoma fibroblasts were more efficient at contracting collagen gels, and displayed altered cell force responses and increased matrix remodelling properties compared to their normal counterparts. Moreover, they were both more responsive to macrophage-derived stimuli and more stimulatory towards macrophages. This pro-fibrotic and pro-inflammatory phenotype was maintained *in vitro* for several passages, indicating a genuine alteration of the resident tissue fibroblasts in trachoma. In agreement with that, we identified a unique pro-inflammatory and pro-fibrotic gene expression signature in scarring trachoma fibroblasts, suggesting that the observed phenotypic differences are underpinned by true differences in gene expression.

### Pro-fibrotic profile of scarring trachoma fibroblasts

Tissue homeostasis is perturbed in fibrotic conditions, where altered matrix expression and remodelling, accompanied by an increased contractility in resident fibroblasts often lead to changes in tissue mechanics and stiffness^22^. This increase in contraction has most often been attributed to myofibroblast differentiation, as defined by a-SMA expression^23^. However, in agreement with our previous work on ocular fibroblasts^9^,^12^,^13^ and other studies^21^,^24^, we found that trachoma-derived fibroblasts did not display a significant increase in a-SMA gene expression, nor do they express the protein in high amounts during contraction *in vitro*. Accordingly, they did not show higher contractile force, whether at resting state or in response to serum, which classically stimulates acto-myosin contraction. Their force response was nevertheless clearly different in terms of dynamics, possibly indicating an adaptation to growth factor stimulation as a result of the long-term chronic stimulation *in vivo*. This is consistent with the expression analysis, which identified various component of the cytoskeleton (both actin and microtubules) and related signalling pathways (including small Rho GTPases regulators) altered in trachoma cells, suggesting that their altered force profile may be due to modified cytoskeletal dynamics downstream receptor activation. GO analysis indicated associations with genes related to muscle (e.g calcitonin-like receptor; dystrophin, Plakophilin), contraction, actin cytoskeleton, cell-cell interactions and cell migration in STFs, possibly supporting the pro-contractile phenotype. Interestingly, it also suggested an apparent differential regulation of microtubules, which has recently been suggested as a way by which Chlamydia infection could reprogram the host cell cytoskeleton^25^. In addition, GO analysis of our dataset partially overlaps with a recent genome-wide association study (GWAS) of scarring trachoma^26^. Thus the trachoma fibroblast signature we identified may reflect a combination of scar-driving alterations following early infection, as well as consequences of the chronic scarring.

When prompted to generate tissue-like structures through contraction of tethered gels, both control fibroblasts and STFs established tensional homeostasis at stiffness levels within the range of that expected of a typical stromal tissue^15^,^27^. However, the elastic modulus of the tissues generated by STFs was significantly higher, potentially mirroring the fibrotic origin of the cells. This may reflect an adaptation to the fibrotic environment^28^, and/or a change in mechanostat level^9^. Noticeably, the stiffness levels reached by STF-derived tissues were still an order of magnitude lower (in the kPa range) than the Elastic modulus values reported for fibrotic tissue, usually in the 10 kPa range and over^29^. This suggests that although alterations to the fibroblasts mechanical properties may contribute to the development of the fibrotic tissue in trachoma, other components, e.g. epithelial layer and inflammation, are needed to recreate the full pathology and subsequent tissue alterations. It is nevertheless possible that because of their altered mechanical properties, the trachoma-derived fibroblasts directly promote fibrosis *in vivo* by sustaining a stiffness-increasing loop, as proposed for lung fibrosis^30^ or cancer^31^. This is supported by the expression array analysis, which revealed alterations in several molecules involved in matrix synthesis and crosslinking in scarring fibroblasts. These include Pro-collagen-lysine, 2-oxoglutarate 5-dioxygenase 2 (*PLOD2*) and collagen modifying molecule leprecan-like 1 (*LEPREL1* or *P3H2*), two enzymes involved in post-translational collagen modification and crosslinking^32^,^33^. Both are significantly upregulated in scarring trachoma fibroblasts (Table S3), and could contribute to matrix stiffening. The array also suggested an upregulation of matrix synthesis functions in scaring fibroblasts, exemplified by an upregulation of collagen type IV (Table S3), as previously observed *in vivo*^34^.

### Pro-Inflammatory profile of scarring trachoma fibroblasts

Scarring trachoma is often accompanied by a sustained inflammatory infiltrate marked by a significant contribution of the innate immune responses, including macrophages. The presence of inflammation is linked to a high recurrence rate following trichiasis surgery^3^,^35^, suggesting that macrophages may contribute to fibroblasts’ stimulation towards scarring. Macrophages did not noticeably affect control or scarring trachoma fibroblast-mediated contraction in the presence of serum, suggesting that the fibroblast’s contractile behaviour is dominated by the response to the biomechanical environment and the mixed growth factor/cytokine stimulation provided by the serum. However, in the absence of serum, macrophages fully stimulated contraction, albeit to a higher degree in scarring trachoma cells, suggesting that diseased fibroblasts interact differently with macrophages. Indeed, our array analysis identified an overall pro-inflammatory profile in STFs, including genes previously implicated in fibrotic conditions and autoimmune disorders such as toll-like receptor 4 (*TLR4*), heme oxygenase 1 (*HMOX1*) and the TNF ligand superfamily member 4 (*TNFSF4*)^36^,^37^, and we were able to show that IL-6 secreted by STFs specifically altered macrophage responses. IL-6 is a pleotropic molecule with diverse roles in health and disease and a driving component in fibrosis^38^,^39^. IL-6 levels are elevated in the conjunctiva mucosal secretions of patients with trachomatous trichiasis, leading to the suggestion that IL-6 may be a prognostic marker for scarring trachoma, but the exact cellular origin of IL-6 in such secretions is unclear^18^. We showed here that IL-6 expression and secretion were up-regulated in STFs, but did not play a direct role in the contraction phenotype, even in the presence of macrophages. Rather, it contributed to an increase in Akt phosphorylation in macrophages, which has been linked to cell survival^20^. Thus, increased IL-6 secretion in trachoma may sustain local macrophage survival and activation, which in turn could further the contraction activity of the fibroblasts. We propose that such a “fibrosis activation feed-back loop” could sustain recurrent scarring in trachoma.

### Cell differentiation and neuronal profile

In addition to pro-fibrotic and pro-inflammatory features, the profiling of scarring trachoma fibroblasts revealed a strong involvement of genes linked to differentiation and developmental processes, including Wnt and TGF-b signalling pathways, confirming previous studies^8^,^26^. These pathways are essential during embryogenesis and development, and their interaction and activation in differentiated cells can result in permanent changes in gene expression leading to disease, including fibrosis^40^,^41^,^42^,^43^. Amongst the genes implicated was *WNT2B*, which has been shown to trigger canonical Wnt signalling responses^41^, as well regulate fibrotic and developmental responses^42^,^43^. Its up-regulation in our dataset (Table S3) might indicate a possible role of canonical Wnt signalling in scarring trachoma. Neuronal differentiation markers were also prominent in our GO analysis as well as in the GWAS study^26^, including Neural Cell Adhesion Molecule 2 (*NCAM2*) and transcription factor activating protein 2 a (*TFAP2A*). NCAM2 is predominantly expressed in the brain and stimulates neurite outgrowth and axonal compartmentalization^44^, while TFAP2A has been implicated in eye development^45^ and neuronal stem cell differentiation of mesenchymal stem cells^46^. This suggests a predisposition for transdifferentiation towards neuronal lineage in scarring trachoma, which may partly be down to the neuronal crest origin of the facial fibroblasts^47^.

## Conclusion

Overall we have identified clear phenotypic and gene expression alterations in scarring trachoma fibroblasts, which may underlie the disease pathology. While overlapping with classical pro-fibrotic and pro-inflammatory pathways, the profile of trachoma fibroblasts bears unique features, possibly linked to early events in the disease progression. Partial overlapping of gene ontology with a published GWAS study^26^ suggests that there might be some contribution of genetic factors to the scarring fibroblasts’ phenotype. Alternatively, some of the expression changes could be due to epigenetic modifications, e.g methylation, following early infection events and/or chronic inflammation. Similarly, the cells’ matrix contraction ability was not directly linked to classical contractile markers (e.g. a-SMA expression) or inflammatory stimulus (e.g. TGFb). Rather, more subtle changes in cell dynamics are likely to underlie the contraction phenotype, and classic pro-inflammatory cytokines may promote the scarring indirectly, through reciprocal interactions with inflammatory cells. Indeed, we have identified an IL-6-driven pro-fibrotic/pro-inflammatory feedback loop that may contribute to chronic scarring in trachoma, possibly underlying the recently uncovered link between clinical inflammation and progressive scarring^3^. This suggests that conjunctival stroma may play a more central role than previously thought in the development of the disease and opens new avenues in the design of novel therapeutic targets in scarring trachoma.

## Materials and Methods

Detailed Material and Methods and reagents are provided in the SI Appendix, *Materials and Methods*.

### Cells

Primary fibroblasts were expanded from biopsies obtained from the upper tarsal conjunctiva of Tanzanian patients undergoing trichiasis surgery and control individuals undergoing cataract surgery. This study adhered to the tenets of the Declaration of Helsinki, and was approved by the Tanzanian National Institute of Medical Research, the Kilimanjaro Christian Medical Centre, and the London School of Hygiene and Tropical Medicine Ethics Committees. The study was explained to potential study participants and written informed consent was obtained before enrolment. The cells were maintained in Dulbecco’s modified Eagle’s medium (DMEM) supplemented with 10% (v/v) heat-inactivated foetal bovine serum, 100 IU/ml penicillin, 100 μg/ml streptomycin. Differentiated macrophages were obtained from the U937 cell line following treatment with 100 nM PMA for 3 days. For siRNA experiments, fibroblasts were transfected with siRNAs against IL-6 (ID numbers M-007993-02-0005, Darmacon) using Hiperfect transfection reagent.

### Collagen contraction

Fibroblast-mediated matrix contraction was assessed using free-floating collagen gel lattices, as described before^13^. To determine cell response to cytokines and growth factors, gels were made using serum free (SF) medium with the addition of one of the following factors: IL-1β, PDGF-BB, IL-17A (10 ng/ml, R&D Systems, UK), TGF-β1, TNF-α, CTGF or CXCL5 (5 ng/ml, Peprotech, UK), IL-6 (20 ng/ml, R&D Systems, UK). For co-culture experiments, macrophages were added to the gel mix at a final ratio of 1 (fibroblast): 4 (macrophages). Cell metabolic activity in gels was measured using 10% Alamar blue. MMP activity was measured at day 5 using an MMP activity assay kit according to manufacturer guidelines (Abcam, UK). Supernatants were either incubated for 2 hours with 2 mM APMA (4-aminophenylmercuric acetate) to allow activation of pro-MMPs for total MMP activity or used immediately to measure only active MMPs. An enzyme immunoassay (TaKara BIO INC.) was used to determine Pro-collagen Type I C-peptide release.

### Force Measurements

Cell and Matrix force were calculated using the Palpator^TM^ Force measurement platform (InvivoSciences LLC, McFarland, WI)^14^. Gels were made as described elsewhere^14^ using 4 × 10^5^ cells/ml. The collagen-cell mixture was poured into each well of an eight-well polycarbonate mould (8-well MC-8™ chamber). The matrices were then cultured in complete DMEM at 37 °C for 2 days and starved in serum free medium for one day^14^. Force measurements were taken every 15 min before and after stimulation with 20% FBS for a total of 45 min. A custom Matlab^®^ algorithm^14^ was used to process and analyse the force data to report a numerical parameter that is indicative of the active cell force in the tissue.

### RNA Isolation and Affymetrix GeneChip Processing

RNA extraction was performed on 4 trachoma-derived (F07, F10, F11, F12) and 4 control (C24, C26, C29, C30) fibroblast cell lines (SI Apendix, Table S1) in standard cultures at passages 3 to 6, using an RNeasy kit (Qiagen, UK), with 2 from each group done in independent duplicates (F07, F10 and C26, C30). The resulting RNA samples were then processed and analysed on a cDNA microarray platform (GeneChip Human Gene 1.0ST; Affymetrix, Santa Clara, CA) at the UCL Genomics microarray laboratory, Institute of Child Health (London, UK).

### Microarray Analysis and validation

Gene array .cel files were processed using Genespring (GX version 10.01 2100; Agilent), and data analysis was performed using AltAnalyze. Summarization and normalization of the gene array cel. files was performed using the Robust MultiArray Average (RMA) method. Gene expression levels (Ensembl genes) and GO annotation with GO-Elite software were carried out, using default options. Genes differentially expressed were identified using a combination of a >2-fold change in expression and a significance of *P* < 0.05 (Lima’s, Student’s *t*-test). Validation was performed using qRT-PCR on a real-time PCR system (HT7900 Fast Real-Time PCR; Applied Biosystems) using GAPDH as a reference endogenous control. Western blot validation was performed as previously described^11^ using the following antibodies: IL-6, Mouse IgG1 (R&D systems); Akt, p-Akt (Ser473) (D9E) XP, Stat3 (124H6), p-Stat3 (Tyr705) (D3A7) XP^®^ (Cell Signalling). IL-6 secretion was validated using the Human IL-6 Quantitative ELISA Kit (R&D Systems) according to the manufacturers protocol.

## Additional Information

**How to cite this article**: Kechagia, J.Z. *et al.* Fibroblasts profiling in scarring trachoma identifies IL-6 as a functional component of a fibroblast-macrophage pro-fibrotic and pro-inflammatory feedback loop. *Sci. Rep.* **6**, 28261; doi: 10.1038/srep28261 (2016).

## Supplementary Material

Supplementary Information
