# Supplementary material for: Fibroblasts profiling in scarring trachoma identifies IL-6 as a functional component of a fibroblast-macrophage pro-fibrotic and pro-inflammatory feedback loop
Source: Sci Rep. 2016 Jun 20;6:28261. doi: 10.1038/srep28261 (PMC4913315; doi:10.1038/srep28261)
Supplement: Supplementary Information [file srep28261-s1.pdf]

Fibroblasts profiling in scarring trachoma identifies IL6 as a functional component of a fibroblast-macrophage pro-fibrotic and pro-inflammatory feedback loop

Zanetta-Zoi Kechagia, Daniel G. Ezra, Matthew J. Burton and Maryse Bailly

Supplementary Information

Supplementary *Materials and Methods*

Biopsy collection and expansion

Biopsies from cases were collected from adults (age >18 years old) with clearly established Trachomatous Trichiasis with associated scarring of the upper tarsal conjunctiva (Grade 2 or 3 on the detailed WHO FPC Trachoma grading scale), undergoing trichiasis surgery. Patients that had been subjected to previous surgery or had conjunctival scarring from a cause other than trachoma were excluded from the study. Control biopsies were collected from adults without Trachomatous Trichiasis or any clinically apparent conjunctival disease or scarring, which were due to undergo ophthalmic surgery for a condition unrelated to disease of the conjunctiva (eg cataract) and had no previous record of eyelid surgery.

The eyelid was anaesthetized with an injection of 2% lignocaine and the eye cleaned with 5% povidone iodine. A biopsy sample was taken using a 3mm trephine from the tarsal conjunctiva, 2mm from the lid margin, at the junction of the medial and lateral of the everted lid. None of these cases had evidence of current C. *trachomatous* infection as detected by PCR (Amplicor PCR, Roche). The biopsies were either wrapped in sterile gauze and moistened with normal saline or placed in Optimem medium (Life Technologies) and transported to the laboratory at +8°C. Biopsies derived from trachoma and control patients were used for fibroblast expansion, with similar age range and gender for both sets (Table S2). The explants where placed in 32 mm tissue culture plates after being dissected and digested at 37ºC for 10-15min using 0.05% Collagenase in PBS. Cells were then cultured in Dulbecco's modified Eagle's medium (DMEM) (PAA laboratories, Austria or Life Technologies) supplemented with 10% (v/v) heat-inactivated foetal bovine serum (FBS, Sigma-Aldrich, UK), 100 IU/ml penicillin, 100 μg/ml streptomycin (hereafter referred to as complete medium) until 40% confluence and then trypsinised and passaged in a T25 tissue culture flask (Corning, NY, USA). Contaminating epithelial and goblet cells were eliminated from the cultures after the first or second passage. Cultures were assessed for typical fibroblast morphology by phase contrast microscopy before every experiment. Cells were then maintained in complete medium and used between passages 2 and 8 for all the experiments. Serum-free medium was used where indicated with or without the addition of cytokines.

Collagen Contraction assay

Control and diseased fibroblasts were embedded in three-dimensional, collagen type I matrix (First Link Ltd., Birmingham, UK) at a final collagen concentration of 1.4 mg/ml after pH was rapidly adjusted to 7 using 1M NaOH. Cells were added to the collagen mixture at a final concentration 8 x10^4 cells/ml or 16 x 10^4/ml for FBS-stimulated or serum free contraction assays, respectively. For co-culture experiments, macrophages were added to the gel mix at a final ratio of 1 (fibroblast): 4 (macrophages) (8 x10^4 cells/ml : 3.2x10^5 cells/ml) and cultured in 10% FBS or SF DMEM medium. The collagen lattices were cast on Mattek® dishes (MatTek Corporation, Ashland, MA) and after 20 min incubation at 37°C gels were detached and medium was added. Reduction in lattice area at days 1, 3, and 7 due to contraction was digitally photographed, and the gel areas calculated using image analysis software (ImageJ, rsbweb.nih.gov/ij/).

To determine cell response to cytokines and growth factors, gels were made using SF medium (supplemented with 0.7% bovine serum albumin (BSA) (Sigma-Aldrich, UK) and 100 IU/ml penicillin, 100 μg/ml streptomycin (Gibco, Life Technologies, UK) with the addition of one of the following factors: IL-1β, PDGF-BB, IL-17A (10 ng/ml, R&D Systems, UK), TGF-β1, TNF-α, CTGF or CXCL5 (5 ng/ml, Peprotech, UK), IL-6 (20 ng/ml, R&D Systems, UK). The optimal concentration of cytokines and growth factors was first determined after testing a wider range of concentrations.

Collagen gel imaging

Immunofluorescence was performed as described before (1). Briefly, collagen gels were fixed using 3.7% Formaldehyde (Sigma-Aldrich, UK) followed by a 30-minute incubation in 0.5% Triton-X100 (Sigma) and 0.1 M glycine. The gels were then stained with Rhodamine-phalloidin (Invitrogen, Life Technologies, UK) in Tris Buffer Saline (TBS) additioned with 1% BSA and 1% FBS in TBS at various time points during contraction. The gels were then imaged using a Biorad Radiance confocal microscope (Zeiss Axiovert S100/Biorad Radiance 2000; Zeiss, Cambridge, UK) to visualize the cells (red, Green HeNe laser 540⁄565 nm) and the matrix (confocal reflection microscopy) using a long working distance objective (ZEISS LD plan-Neofluoar 63x/0.75).

Elastic modulus calculation

The Elastic Modulus (Young's modulus) of the tissue contracts was calculated from the formula: $Fm = E \times A$, where Fm is the matrix force and A is the cross-sectional area of the gels (2) with $A = Tissue\ heigh \times probe\ length.$ Tissue height was evaluated manually using the focus drive function on a software driven (OpenLab, Improvision) Zeiss Axiovert 100M microscope (Zeiss plan-neofluar 10x/0.30). The length of the probe used for the indentations was 5 mm. For each tissue construct, tissue height was averaged from 5 measurements, and 4 gels per experiment and per condition were used to calculate the elastic modulus.

Flow cytometry

Flow cytometry was used to assess integrin expression of 3 Control and 3 trachoma cell lines. Cells were trypsinised, washed with PBS and incubated with FITC conjugated antibodies for 1h on ice. The conjugated antibodies used are listed in Table S1. Cells were acquired on a BD FACSCalibur using CellQuest (BD Cytometry Systems) at the FACS facility in the Institute of Ophthalmology, UCL. Flow cytometry analyses were performed using FlowJo Software (Tree star, OR USA).

Macrophage differentiation

U937 monocytes were cultured in RPMI1640 (Gibgo, Life technologies, UK) supplemented with 10% FBS, 100 IU/ml penicillin, and 100 μg/ml streptomycin (complete RPMI). For differentiation into macrophages, the cells were incubated with 100 nM PMA for 3 days in complete RPMI, and further rested for 2-3 days in regular complete RPMI.

Metabolic activity

Cell metabolic activity was measured at day 4 of fibroblasts growing in collagen gels. Cells/gels were incubated for 3 hour in 37^0C with 10% Alamar blue in 10% FBS DMEM medium. After 3 hours supernatants were examined for fluorescence absorbance, according to manufacture guidelines.

MMP activity

MMP activity released in the medium during gel contraction was measured at day 5 using an MMP activity kit (MMP Activity Assay Kit, Abcam, UK), following manufacturers instructions.

Pro-collagen synthesis

An enzyme immunoassay (TaKara BIO INC.) was used to determine the Pro-collagen Type I C-peptide concentration in conditioned medium of contracting gels. Supernatants from Day 3 and 7 of contraction were collected and kept in -80 ºC until used. The supernatants from 8 control and 8 cases were examined for Pro-collagen Type I C-peptide concentration, following manufacturer's protocol for higher sensitivity Samples. The % change in pro-collagen I synthesis was calculated for each cell line.

Real time qPCR

Reverse transcription was carried out from the extracted mRNA using the QuantiTect Reverse Transcription Kit (Qiagen, UK) according to manufacturer's instructions. Gene expression was measured by qRT-PCR using premade primers with FAM™ dye-labeled TaqMan® MGB probe (250 nM final 1X reaction concentration) and *Taq* PCR Master Mix Kit (Qiagen, UK) according to the supplied protocol. The primers had the following assay ID: NM_002046.3 (GAPDH), Hs99999032_m1 (IL-6), Hs00189850_m1 (NCAM2), Hs00261096_s1 (PCDH7), Hs01029413_m1 (TFAP2A), Hs00170261_m1 (THBS4) (Assay-on-Demand; Applied Biosystems, Foster City, CA). qRT-PCR reactions were performed on a real-time PCR system (HT7900 Fast Real-Time PCR; Applied Biosystems). Data were analysed using the comparative ΔCT method (3).

Western blot

Western blot was performed as previously described (1). The following antibodies were used in this study: IL-6, Mouse IgG1 (R&D systems); Akt, p-Akt (Ser473) (D9E) XP, Stat3 (124H6), p-Stat3 (Tyr705) (D3A7) XP® (Cell Signalling).

References

1. Tovell VE, Chau CY, Khaw PT, Bailly M (2012) Rac1 inhibition prevents tissue contraction and MMP mediated matrix remodeling in the conjunctiva. *Invest Ophthalmol Vis Sci* 53(8):4682–91.
2. Marquez JP, et al. (2009) High-throughput measurements of hydrogel tissue construct mechanics. *Tissue Eng Part C Methods* 15(2):181–90.
3. Livak KJ, Schmittgen TD (2001) Analysis of relative gene expression data using real-time quantitative PCR and the 2(-Delta Delta C(T)) Method. *Methods* 25(4):402–8.

Supplementary *Figure Legends*

Figure S1. Control and trachoma derived cells exhibit a similar morphology in culture. (A) Shown are 5 representative control and trachoma-derived fibroblast cultures after expansion (passage 5-7). Scale bar, 100 μm. **(B-C)** Analysis of the expression profile of integrins β1, β2, β4, αv, α3 and α4 was performed using FACS. CFs and STFs have similar integrin expression levels, both in terms of *(B)* percentage of positive cells and *(C)* levels of expression (mean fluorescence intensity, MFI). Both b1 and aV integrins are highly expressed in CFs and STFs. Graphs show the mean +/- SEM for 3 STFs and 3 CFs from 2 independent experiments.

Figure S2. Trachoma fibroblasts display increased matrix degradation during contraction. Trachoma and control fibroblasts were seeded in collagen matrices for contraction in presence of 10% serum and the gels were fixed and stained with Rhodamine phalloidin at day 1, 3 and 7 of contraction. The gels were then imaged using a confocal microscope and representative z-sections were captured for each time point. F-actin: red, maximum intensity projection; matrix: confocal reflection microscopy, one representative confocal slice (midway through the cell volume). Shown are representative images from ***(A)*** one CF (C29) and ***(B)*** one STF (F07). Arrows indicate distinct collagen fibres, noticeably more abundant in the gels populated with CFs. Haze denotes partially degraded collagen. Scale bar= 40μm.

Figure S3. a-SMA expression in contracting fibroblasts. Trachoma and control fibroblasts were seeded in collagen matrices for contraction in presence of 10% serum and the gels were fixed and stained for a-SMA and F-actin at day 7 of contraction. The gels were then imaged using a confocal microscope to visualize the cells and the percentage of a-SMA positive cells was determined on 3 CFs (C29, C26, C31) and 3 STFs (F10, F99, F106) cell lines. ***(A)*** A representative example of an a-SMA positive (right) and a negative (left) cells (F-actin, red; α-SMA, green). Scale bar, 40 μm. ***(B)*** Proportion of α-SMA positive cells at day 7 of contraction in 3D collagen matrices. Shown is the mean +/- SEM of 3 independent experiments for 3 CFs and 3 STFs.

Figure S4. *(A)* Modulation of IL-6 activity or expression. IL-6 neutralizing antibody (IL-6 nAb) activity cause a dose depended decrease in STAT3 phosphorylation in macrophages. siRNA treatment downregulate IL-6 expression in fibroblasts. ***(B)***

Macrophages alone do not contract collagen gels. Macrophages alone cultured in gels in (i) medium containing 10% serum or (ii) in serum-free medium did not contract collagen gels. Shown are macrophage-containing gels after 7 days culture. The outer circle denotes the size of the gels at the start of the experiment. ***(C)*** **IL-6 stimulates STAT3 and Akt phosphorylation in macrophages in a dose- and time- dependent manner.** Lysates from macrophages treated with different concentrations of IL-6 (0, 10, 20, 40 ng/ml) in serum free medium indicate a dose response to IL-6 stimulation, as indicated by STAT3 and Akt phosphorylation levels. ***(D)*** **CFs and STFs differentially activate Akt phosphorylation in macrophages through an IL-6 dependent manner.** CFs and STFs-derived CM, obtained from fibroblasts treated with non-target control siRNA (NT control) or IL-6 targeting siRNA (IL-6 siRNA), was added to U937 differentiated macrophages. The cells were lysed after 24h incubation and tested for Akt phosphorylation (n=2 for 5 CFs and 5 STFs).

Kechagia et al Supplementary Figure S1

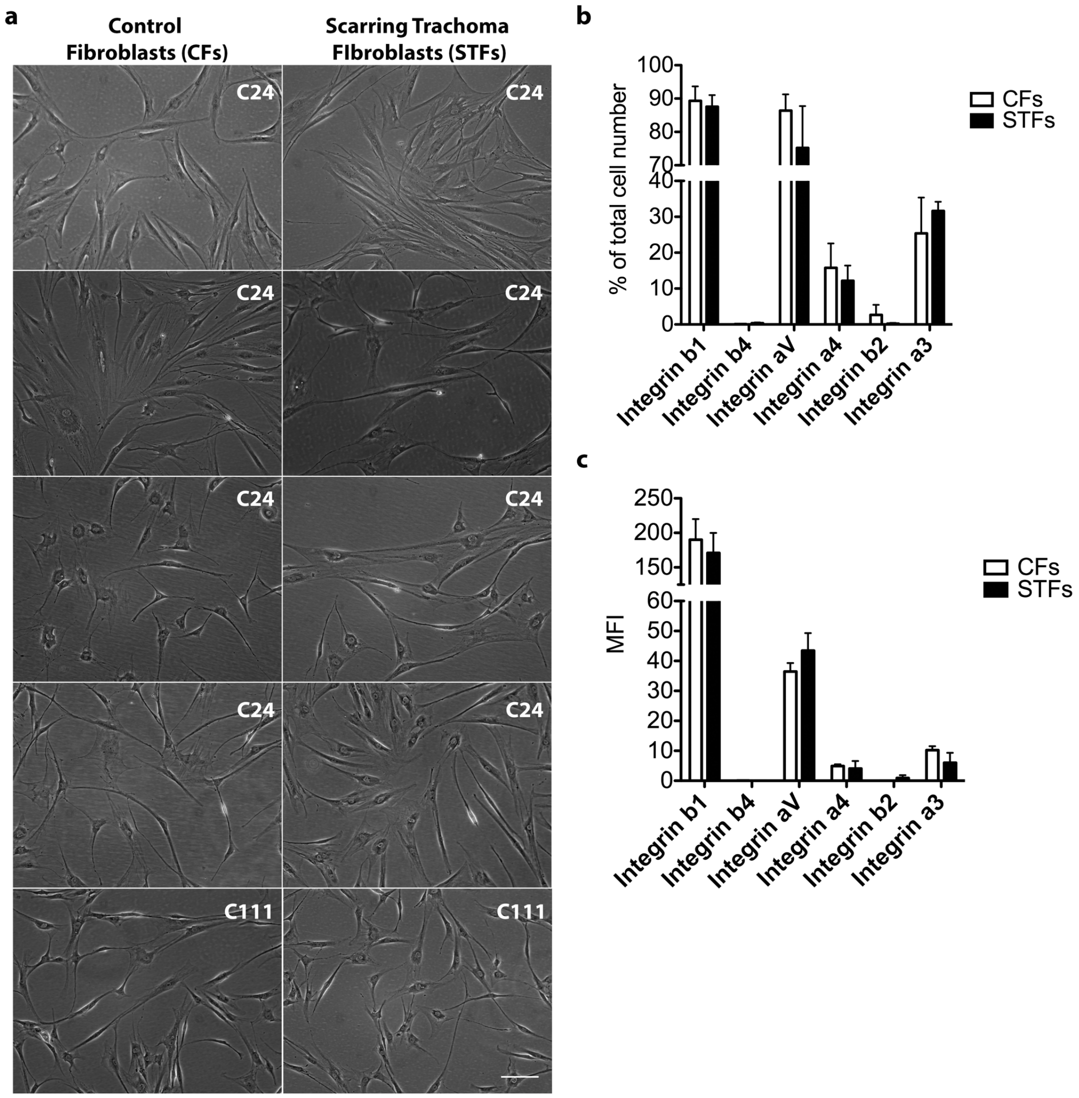

Kechagia et al Supplementary Figure S2

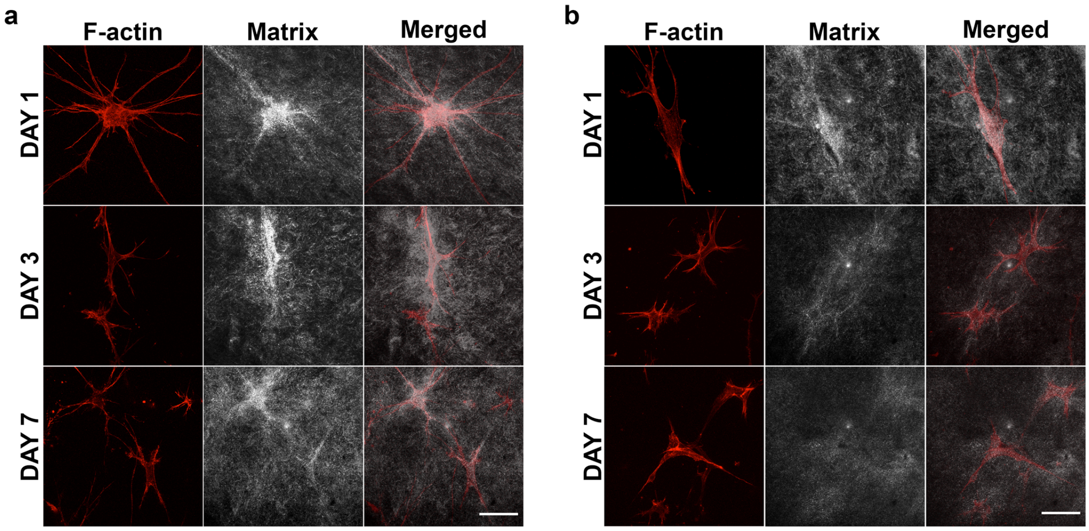

Kechagia et al Supplementary Figure S3

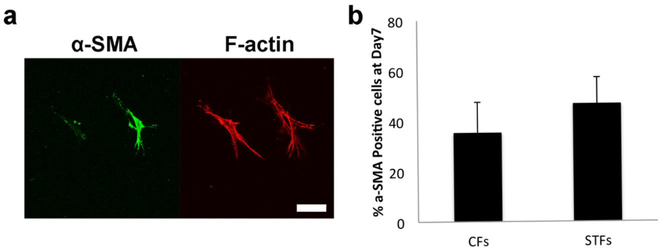

Kechagia et al Supplementary Figure S4

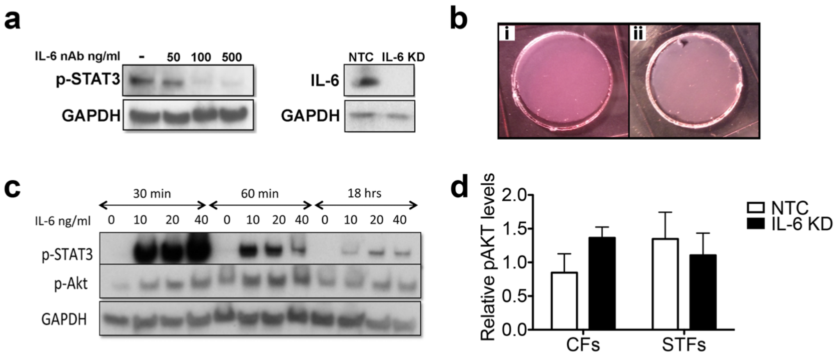

Table S1 *FACS analysis antibodies*

**Antibodies**	**Integrin/FC controls**	**Company**
Alexa Fluor® 488 Mouse IgG1, ϰ Isotype Ctrl (FC)	FC Control	BioLegend, UK
Alexa Fluor® 488 anti-human CD29 Antibody	Integrin β1	BioLegend, UK
FITC Mouse IgG2a, ϰ Isotype Ctrl (FC)	FC Control	BioLegend, UK
FITC anti-human CD104 Antibody	Integrin β4	BioLegend, UK
FITC anti-human CD51 Antibody	integrin αV	BioLegend, UK
FITC Mouse IgG1, ϰ Isotype Ctrl (FC)	FC Control	BioLegend, UK
FITC anti-human CD49d Antibody	α4 integrin	BioLegend, UK
FITC anti-human CD18 Antibody	Integrin β2	BioLegend, UK
Anti-Integrin alpha 3 antibody [17C6] (FITC)	Integrin α3	Abcam
Anti-Integrin beta 3 antibody [C17] (FITC)	Integrin β3	Abcam

Table S2. *Details of the collected biopsies and derived fibroblast cultures*.

Study number	Case or control*	Sex**	Age (years)	Ethnic Group¶	Expanded cell line name
001	0	2	63	5	-
003	0	1	75	10	C03
005	0	1	77	2	-
006	0	1	62	10	C06
020	0	1	17	5	-
021	0	1	87	5	-
022	0	2	81	11	-
024	0	1	-	2	C24
025	0	2	-	2	-
026	0	2	60	2	C26
027	0	2	-	2	-
028	0	1	70	1	-
029	0	2	33	2	C29
030	0	1	99	2	C30
031	0	2	52	1	C31
092	0	2	78	7	-
100	0	1	64	7	-
103	0	1	86	2	C103
110	0	2	62	2	C110
111	0	2	70	2	C111
F-02	1	2	73	2	F02
F-04	1	2	80	2	-
F-07	1	1	77	2	F07
F-09	1	1	50	2	F09
F-10	1	2	72	2	F10
F-11	1	2	72	2	F11
F-12	1	2	70	2	F12
F-13	1	2	68	2	F13
F-14	1	2	-	2	F14
F-15	1	2	79	2	-
F-23	1	2	58	1	-
098	1	2	40	2	-
090	1	2	80	1	-
091	1	2	52	1	-
093	1	2	77	2	-
095	1	2	70	2	-
096	1	2	28	2	F96
099	1	1	35	2	F99
101	1	1	60	1	-
102	1	2	65	1	-
104	1	1	70	2	-
105	1	1	62	1	-
106	1	2	70	2	F106
107	1	-	-	-	-
108	1	-	-	-	-
109	1	-	-	-	F109

*: 0= control and 1= case; **: 1=male; 2=female; ¶: 1= Masai; 2= Chagga; 5 = Pare; 7= Mzigua; 10= Samba; 11= Other

Table S3. *Differentially regulated genes (moderate t-test p<0.05 and FC>± 2) in STFs, compared to CFs. Rawp values are generated using limma's moderate t-test.*

Symbol	fold-STF_vs_CF	rawp-STF_vs_CF	Ensembl_gene
NCAM2	11.73103785	4.62E-05	ENSG00000154654
TFAP2A	10.52773008	0.001381404	ENSG00000137203
PDE5A	9.119327895	0.014512128	ENSG00000138735
OLR1	6.780713474	0.015470846	ENSG00000173391
IL6	6.182493209	0.009692134	ENSG00000136244
LIMCH1	5.921673715	0.006130807	ENSG00000064042
TMEM176B	5.775186353	0.001015357	ENSG00000106565
NXN	5.751799289	0.004531278	ENSG00000167693
TNS3	5.341687675	0.000358491	ENSG00000136205
STMN2	5.325557278	0.021171171	ENSG00000104435
MAOA	4.534871486	0.001240538	ENSG00000189221
SMOC2	4.533703364	0.026238629	ENSG00000112562
SEMA3D	4.521037515	0.027926293	ENSG00000153993
EYA4	4.489393716	0.016403405	ENSG00000112319
TMEM176A	4.388787749	0.000598219	ENSG00000002933
TNFSF4	4.31466186	0.012499209	ENSG00000117586
BCHE	4.27748783	0.001510589	ENSG00000114200
AL354993.1	4.151654103	0.017272168	ENSG00000227164
GPR133	4.104915431	0.037620594	ENSG00000111452
PTGER3	4.090649705	0.012247946	ENSG00000050628
OXTR	3.900945249	0.033342103	ENSG00000180914
PALMD	3.900090226	0.019165553	ENSG00000099260
CLIC6	3.782258911	0.023516803	ENSG00000159212
ENTPD1	3.7204574	0.007779821	ENSG00000138185
TSHZ2	3.61673401	0.039408852	ENSG00000182463
GATA6	3.593801769	0.00634203	ENSG00000141448
DUXA	3.590114856	0.004667516	ENSG00000258873
MPP7	3.558329229	0.039179823	ENSG00000150054
KLRD1	3.485885059	0.007360197	ENSG00000134539
PARD6G	3.48073022	0.000585283	ENSG00000178184
PKP2	3.473141647	0.035593806	ENSG00000057294
AP1S3	3.438966352	0.010830412	ENSG00000152056
DMD	3.374604274	0.010098275	ENSG00000198947
CNTN1	3.342071272	0.026649684	ENSG00000018236
LUZP2	3.305988409	0.000813787	ENSG00000187398
CTSH	3.304182408	0.000435221	ENSG00000103811
LEPR	3.274010995	0.000401211	ENSG00000116678
LYPD6B	3.221471876	0.041252745	ENSG00000150556
ADARB1	3.206456866	0.006493234	ENSG00000197381
PCDHB3	3.178027628	0.002928496	ENSG00000113205
CDH8	3.145106237	0.009473557	ENSG00000150394
LRRC15	3.104153714	0.026241295	ENSG00000172061
POLR3G	3.077342456	0.000807768	ENSG00000113356
CALCRL	2.976821055	0.00630944	ENSG00000064989

GGT5	2.976179759	0.005367803	ENSG00000099998
LEPREL1	2.972261054	0.015186769	ENSG00000090530
DEPTOR	2.924064226	0.006346508	ENSG00000155792
RARRES1	2.889785681	0.043547804	ENSG00000118849
RDH5	2.888696729	0.001341345	ENSG00000135437
CHST15	2.859987319	0.005943409	ENSG00000182022
PRKG2	2.84129647	0.006231375	ENSG00000138669
C10orf10	2.832179098	0.015042242	ENSG00000165507
LYPD6	2.81833707	0.014422166	ENSG00000187123
FAM107B	2.795652898	0.010379882	ENSG00000065809
KCND2	2.790315649	0.002192635	ENSG00000184408
RRAGD	2.766783964	0.004281383	ENSG00000025039
FGF1	2.720999139	0.014985931	ENSG00000113578
COL4A2	2.644651274	0.00581833	ENSG00000134871
SLC16A3	2.624822744	0.023776676	ENSG00000141526
LIPH	2.609554893	0.014754145	ENSG00000163898
RASGRF2	2.594611637	0.020097152	ENSG00000113319
CRISPLD2	2.57390713	0.020867896	ENSG00000103196
UPK1A	2.557020098	0.049508053	ENSG00000105668
HSPB8	2.553219374	0.022500189	ENSG00000152137
TFAP2A-AS1	2.552998164	0.043332356	ENSG00000229950
SPOCD1	2.493440021	0.00824046	ENSG00000134668
PMEPA1	2.482426925	0.014754014	ENSG00000124225
RNF180	2.466374138	0.005637032	ENSG00000164197
SEMA6A	2.461306739	0.042658446	ENSG00000092421
JUP	2.449131096	0.004285231	ENSG00000173801
FILIP1L	2.448117591	0.016437802	ENSG00000168386
BDH1	2.447154181	0.032145625	ENSG00000161267
SESN3	2.435123598	0.008145962	ENSG00000149212
DMRTA1	2.415166247	0.000235437	ENSG00000176399
ANKRD6	2.413898475	0.008454045	ENSG00000135299
WNT2B	2.397100672	0.00352652	ENSG00000134245
ALPL	2.38794151	0.045155748	ENSG00000162551
PCDHGA10	2.376424389	0.024457636	ENSG00000253846
MAP6	2.345978839	0.031290271	ENSG00000171533
WIPF3	2.345575395	0.010778569	ENSG00000122574
TMEM130	2.344286264	0.047202066	ENSG00000166448
ST3GAL6	2.340806871	0.010958331	ENSG00000064225
FLT1	2.324889396	0.000213086	ENSG00000102755
PCDH7	2.317601462	0.000783016	ENSG00000169851
UCHL1	2.316358144	0.00087477	ENSG00000154277
KIF20A	2.29717179	0.002719552	ENSG00000112984
ID3	2.288879479	0.001011283	ENSG00000117318
TSPAN12	2.281956719	0.001851277	ENSG00000106025
FGD4	2.268875585	0.02666006	ENSG00000139132
APBB1IP	2.247855946	0.003582197	ENSG00000077420
RASSF4	2.228373786	0.030497019	ENSG00000107551
UGT1A7	2.227298624	0.000161648	ENSG00000244122

CCNB1	2.220556839	0.000788793	ENSG00000134057
PCDH10	2.200589426	0.017481848	ENSG00000138650
PRR5L	2.194670311	0.020981069	ENSG00000135362
EPAS1	2.194465174	0.003728483	ENSG00000116016
TLR4	2.192703892	0.022561924	ENSG00000136869
GUCY1A3	2.189846127	0.019809201	ENSG00000164116
PLXDC2	2.142474046	0.016835382	ENSG00000120594
OSGIN2	2.136847202	0.011071153	ENSG00000164823
MRVI1	2.134976219	0.0293845	ENSG00000072952
LPPR4	2.129216687	0.031200783	ENSG00000117600
SDPR	2.12174404	0.0065971	ENSG00000168497
DPM2	2.11405684	0.00959718	ENSG00000136908
DNAJC6	2.110174325	0.004672611	ENSG00000116675
PCDHB4	2.104890232	0.045291625	ENSG00000081818
TMOD1	2.104709081	0.025962297	ENSG00000136842
FBLN7	2.102655503	0.023663858	ENSG00000144152
FAM20A	2.090022297	0.031908696	ENSG00000108950
HAP1	2.089582906	0.029686781	ENSG00000173805
NCAPH	2.084799655	0.046976262	ENSG00000121152
CEP55	2.077852326	0.021860868	ENSG00000138180
SLC4A4	2.073165082	0.032932318	ENSG00000080493
TINAGL1	2.071448568	0.019801666	ENSG00000142910
PAG1	2.071033419	0.003271356	ENSG00000076641
GGH	2.061256438	0.008519441	ENSG00000137563
SULT1B1	2.058373801	0.03708697	ENSG00000173597
PCDHB2	2.040304057	0.037754186	ENSG00000112852
FAM213A	2.036073123	0.005287627	ENSG00000122378
MCOLN3	2.032978559	0.00867724	ENSG00000055732
SULF2	2.03209652	0.028371138	ENSG00000196562
PIEZO2	2.029576347	0.003481317	ENSG00000154864
PLOD2	2.027186204	0.005910056	ENSG00000152952
PLK1	2.020354911	0.015713003	ENSG00000166851
FLRT2	2.018536955	0.022351759	ENSG00000185070
SLC24A3	2.012010034	0.006938288	ENSG00000185052
KIF11	2.007167826	0.006021509	ENSG00000138160
LGR4	2.002136033	0.007188473	ENSG00000205213
PLK1S1	-2.022300657	0.00013216	ENSG00000088970
KIF6	-2.076633566	0.009413183	ENSG00000164627
PPL	-2.077859528	0.003361394	ENSG00000118898
C1QTNF1	-2.083382159	0.001865598	ENSG00000173918
HMOX1	-2.084526915	0.000659409	ENSG00000100292
ST6GALNAC3	-2.092343909	0.008759943	ENSG00000184005
HIST1H3E	-2.119459282	0.016251148	ENSG00000196966
KCNT2	-2.126074604	0.041856407	ENSG00000162687
CLEC3B	-2.127910541	0.03736542	ENSG00000163815
DRP2	-2.131968689	0.009578446	ENSG00000102385
PPP1R1C	-2.180467519	0.00240958	ENSG00000150722
MSX1	-2.210461336	0.049357291	ENSG00000163132

NOG	-2.264386143	0.035265016	ENSG00000183691
GPC6	-2.275145403	0.038473033	ENSG00000183098
TIMP4	-2.281397758	0.04805156	ENSG00000157150
COL21A1	-2.305602698	0.019008653	ENSG00000124749
CIITA	-2.307978845	0.009671071	ENSG00000179583
ATF5	-2.327244035	0.004882409	ENSG00000169136
NDUFA4L2	-2.346743234	0.002704909	ENSG00000185633
CA12	-2.369762023	0.043816423	ENSG00000074410
CORO2B	-2.389740488	0.031240666	ENSG00000103647
OLFML2A	-2.390311684	0.009787756	ENSG00000185585
EBF3	-2.406062779	0.015779339	ENSG00000108001
FAM129A	-2.529731906	0.000440262	ENSG00000135842
SV2B	-2.533992931	0.007398191	ENSG00000185518
ANKFN1	-2.537560994	0.006498238	ENSG00000153930
ROBO3	-2.548630983	0.005595371	ENSG00000154134
PTPRD	-2.562021353	0.013103748	ENSG00000153707
COCH	-2.654913763	0.007324238	ENSG00000100473
TMEM255B	-2.76460813	0.038999626	ENSG00000184497
IL16	-2.894583618	0.04203362	ENSG00000172349
ARHGAP26	-2.903540434	0.000665198	ENSG00000145819
SERPINA3	-2.915118727	0.017890377	ENSG00000196136
B3GALT1	-2.941521335	0.027684912	ENSG00000172318
CHI3L2	-3.072589426	0.004567157	ENSG00000064886
COLEC12	-3.089181207	0.010828652	ENSG00000158270
CCRL1	-3.222281424	0.004033437	ENSG00000129048
TM4SF1	-3.635311945	0.006795721	ENSG00000169908
IL31RA	-3.664012372	0.030979677	ENSG00000164509
PAPPA2	-3.670680383	0.016878892	ENSG00000116183
CPM	-4.18300218	0.005003751	ENSG00000135678
THBS4	-6.636389211	0.008300084	ENSG00000113296
WISP3	-6.657278393	0.002054938	ENSG00000112761
SCRG1	-7.97015897	0.000297917	ENSG00000164106
MYOC	-8.213853219	0.034851036	ENSG00000034971
RGCC	-9.15011579	0.013851614	ENSG00000102760

Table S4. Gene ontology analysis (biological processes) of differentially expressed genes, as predicted by ORA analysis in GO-Elite (z-score> 2, Fisher's exact p_{value}< 0.05).

Ontology Name	Z Score	Fisher Exact P	gene symbols
**Pro-Inflammatory**			
negative regulation of cytokine secretion	5.61	0.0018	IL-6, RGCC, TNFSF4
acute inflammatory response	5.11	0.0014	IL-6, PTGER3, SERPINA3, TNFSF4
positive regulation of interleukin-6 production	4.51	0.0051	IL-6, TLR4, TNFSF4
response to antibiotic	4.51	0.0051	ALPL, CIITA, IL-6
positive regulation of inflammatory response	4.11	0.0045	IL-6, PTGER3, TLR4, TNFSF4
negative regulation of leukocyte activation	3.82	0.0045	HMOX1, IL31RA, PAG1, PDE5A, TNFSF4
positive regulation of B cell activation	3.70	0.0117	IL-6, TLR4, TNFSF4
response to lipopolysaccharide	3.06	0.0112	ALPL, IL-6, PTGER3, TIMP4, TLR4, TNFSF4
positive regulation of immune effector process	2.83	0.0233	HMOX1, IL-6, TLR4, TNFSF4
response to hydrogen peroxide	2.72	0.0350	HMOX1, IL-6, OLR1
immune effector process	2.25	0.0364	ADARB1, CTSH, IL-6, POLR3G, RGCC, TLR4, TNFSF4
regulation of angiogenesis	7.51	1.07E-06	COL4A2, CTSH, FGF1, FLT1, GATA6, HMOX1, IL-6, RGCC, THBS4, TSPAN12
cellular response to hypoxia	5.43	0.0005	CCNB1, EPAS1, GATA6, HMOX1, RGCC
angiogenesis	3.69	0.0033	CALCRL, COL4A2, EPAS1, FGF1, FLT1, HMOX1, TSPAN12
**Pro-fibrotic**			
extracellular structure organization	5.14	0.0001	COL21A1, COL4A2, CRISPLD2, LEPREL1, OLFML2A, PCDHB2, PCDHB3, PCDHB4, PLOD2, SMOC2, SULF2, TFAP2A
regulation of collagen biosynthetic process	6.38	0.0009	CIITA, IL-6, RGCC
calcium-dependent cell-cell adhesion	6.10	0.0012	PCDHB2, PCDHB3, PCDHB4
regulation of cell migration	4.05	0.0009	ADARB1, CTSH, FGF1, FLT1, HMOX1, IL-6, NOG, PKP2, RGCC, TFAP2A, THBS4
muscle cell development	3.58	0.0134	CCNB1, DMD, UCHL1
positive regulation of locomotion	3.32	0.0063	CTSH, FGF1, FLT1, IL16, IL-6, TFAP2A, THBS4
microtubule-based movement	3.27	0.0100	HAP1, KIF11, KIF20A, KIF6, UCHL1
extracellular region part	8.79	2.93E-12	ALPL, BCHE, C1QTNF1, CDH8, CEP55, CHI3L2, CLEC3B, COCH, COL21A1, COL4A2, COLEC12, CRISPLD2, CTSH, ENTPD1, FBLN7, FGF1, FLRT2, FLT1, GGH, GPC6, HMOX1, IL16, IL-6, LEPREL1, LIPH, MYOC, NOG, OLFML2A, PLOD2, SCRG1, SMOC2, SULF2, THBS4, TIMP4, TINAGL1, TNFSF4, WISP3, WNT2B
desmosome	6.10	0.0012	JUP, PKP2, PPL
actin cytoskeleton	3.10	0.0105	CORO2B, DMD, FGD4, HAP1, JUP, SLC16A3

cell-substrate junction	2.43	0.0394	APBB1IP, ARHGAP26, DMD, TNS3
**Differentiation**			
developmental induction	5.61	0.0018	FGF1, TFAP2A, WNT2B
morphogenesis of embryonic epithelium	5.50	0.0020	JUP, NOG, TFAP2A
face morphogenesis	5.50	0.0020	MSX1, NOG, TFAP2A
nervous system development	4.49	0.0004	ARHGAP26, JUP, NOG, PCDHB2, PCDHB3, PCDHB4, SCRG1, SEMA3D, SEMA6A, TFAP2A
canonical Wnt receptor signaling pathway	2.88	0.0292	JUP, LGR4, WNT2B
kidney development	2.78	0.0251	CTSH, ID3, SULF2, TFAP2A
central nervous system development	2.76	0.0256	DRP2, ID3, NOG, TIMP4
regulation of canonical Wnt receptor signaling pathway	2.67	0.0288	ANKRD6, JUP, NOG, WNT2B
heart development	2.57	0.0276	CALCRL, ID3, MSX1, OXTR, PKP2
negative regulation of cell differentiation	2.55	0.0192	ATF5, ID3, IL-6, JUP, MSX1, NOG, PKP2, TLR4, TNFSF4
neuron differentiation	2.24	0.0444	ID3, MCOLN3, PTPRD, STMN2, WNT2B

Table S5 Pathway analysis of differentially regulated genes (STFs_vs_CFs) based on Wikipathay and KEGG pathway databases as predicted by GO-Elite software (Fisher Extract p_{value}<0.05 and z_score > 2).

Gene-Set Name	Percent Changed	Z Score	Fisher Exact P	gene symbols
**Differentiation Pathway** (WP2848)	8.5106	4.9519	0.0015	FGF1, IL-6, NOG, WNT2B
**Malaria** (KEGG-hsa05144)	4.6875	3.2812	0.0184	IL-6, THBS4, TLR4
**Arrhythmogenic right ventricular cardiomyopathy** (KEGG-hsa05412)	3.4091	2.5608	0.0418	DMD, JUP, PKP2
**TGF-beta signalling pathway** (KEGG-hsa04350)	3.2258	2.4428	0.0479	ID3, NOG, THBS4
